# Engrafted human cells generate adaptive immune responses to Mycobacterium bovis BCG infection in humanized mice

**DOI:** 10.1186/1471-2172-14-53

**Published:** 2013-12-07

**Authors:** Jinhee Lee, Michael A Brehm, Dale Greiner, Leonard D Shultz, Hardy Kornfeld

**Affiliations:** 1 Department of Medicine, University of Massachusetts Medical School, Worcester, MA, USA; 2 Department of Molecular Medicine, University of Massachusetts Medical School, Worcester, MA, USA; 3 The Jackson Laboratory, Bar Harbor, ME, USA

**Keywords:** Animal model, BCG, Tuberculosis, BLT mice, NSG mice

## Abstract

**Background:**

Currently used mouse models fail to fully reflect human immunity to tuberculosis (TB), which hampers progress in research and vaccine development. Bone marrow-liver-thymus (BLT) mice, generated by engrafting human fetal liver, thymus, and hematopoietic stem cells in severely immunodeficient NOD/SCID/IL-2Rγ^-/-^ (NSG) mice, have shown potential to model human immunity to infection. We engrafted HLA-A2-positive fetal tissues into NSG mice transgenically expressing human leukocyte antigen (HLA)-A2.1 (NSG-A2) to generate NSG-A2-BLT mice and characterized their human immune response to *Mycobacterium bovis* bacillus Calmette-Guerin (BCG) infection to assess the utility of this model for investigating human TB.

**Results:**

NSG-A2-BLT mice were infected intravenously with BCG and the immune response of engrafted human immune cells was characterized. After ex vivo antigenic stimulation of splenocytes, interferon (IFN)-γ-producing cells were detected by ELISPOT from infected, but not uninfected NSG-A2-BLT mice. However, the levels of secreted IFN-γ, determined by ELISA, were not significantly elevated by antigenic stimulation. NSG-A2-BLT mice were susceptible to BCG infection as determined by higher lung bacillary load than the non-engrafted control NSG-A2 mice. BCG-infected NSG-A2-BLT mice developed lung lesions composed mostly of human macrophages and few human CD4^+^ or CD8^+^ T cells. The lesions did not resemble granulomas typical of human TB.

**Conclusions:**

Engrafted human immune cells in NSG-A2-BLT mice showed partial function of innate and adaptive immune systems culminating in antigen-specific T cell responses to mycobacterial infection. The lack of protection was associated with low IFN-γ levels and limited numbers of T cells recruited to the lesions. The NSG-A2-BLT mouse is capable of mounting a human immune response to *M. tuberculosis* in vivo but a quantitatively and possibly qualitatively enhanced effector response will be needed to improve the utility of this model for TB research.

## Background

Tuberculosis (TB), caused by *Mycobacterium tuberculosis* (Mtb), remains a serious human health threat, killing 1.4 million people annually (Tuberculosis Fact Sheet, 2011, World Health Organization, Geneva, Switzerland). Despite numerous TB vaccine candidates being tested, the prospects for their success have dimmed following the failure of the MV85A TB vaccine in a recent clinical trial [1]. A major obstacle to TB vaccine development is the incomplete understanding of the human immune response to TB and the correlates of protection.

Animal models, including mice, guinea pigs, rabbits and non-human primates have contributed to our current knowledge on Mtb-host interactions. The availability of genetically modified mice enabled researchers to identify crucial components of protective immunity including interferon (IFN)-γ, tumor necrosis factor (TNF)-α, and interleukin (IL)-12 [2]. The roles of these factors have been verified in humans with mutations in the IFN-γ or IL-12 receptor genes and in patients treated with TNF-α blockers [3,4]. Despite the usefulness of traditional animal models, they are criticized for not accurately reflecting human TB. The most critical difference lies in their homogenous susceptibility; 100% of mice, rabbits and guinea pigs infected with Mtb develop disease and eventually die with progressive lung pathology, while only 5-10% of Mtb-infected humans develop active TB disease [5]. Most infected individuals mount an effective immune response that maintains latency throughout their lifespan [5]. Latent TB infection is a unique feature of human TB, for which there is no optimal animal model. Non-human primates are capable of enforcing latency but nonetheless have a much higher rate of TB progression than humans, while rodent models are incapable of enforcing latency [6]. Another characteristic feature of human TB is the formation of organized, caseating granulomas with a macrophage core and a peripheral rim of lymphocytes [7]. Although TB lesions in mice contain macrophages and lymphocytes, they are poorly organized and generally not necrotic. Non-human primate (NHP) models mirror human TB pathology, but the use of NHP is limited by high cost and the lack of genetic models. These considerations emphasize the need for new and more tractable animal models that better reflect the unique features of human immunity to TB.

Recent advances in humanized mice hold promise for an enhanced model of human immunity. Successful human cell engraftment requires an immunodeficient recipient. Advances in humanized mouse models have followed the generation of mouse strains with increasingly broader deficiency in host innate immunity and expression of human-specific cytokines and other factors. Attempts to engraft mice with human hematopoietic cells began with CB-17-*Prkdc*^*scid*^ , severe combined immune deficiency, (SCID) mice as recipients, but results were limited by the development of functional mouse lymphocytes, moderate NK cell activity, and only limited engraftment [8]. Subsequently, the *scid* mutation was backcrossed onto the NOD strain and combined with the *IL2rg*^*Tm1Wjl*^ (IL2rγ^null^) mutation to create the NOD-SCID*-*IL2rγ^null^ strain [9]. The most advanced engraftment iteration of this model employs mice engrafted with human fetal thymus and liver followed by transplantation of autologous CD34^+^ hematopoietic stem cells (HSC), termed BLT (bone marrow-liver-thymus) mice [10]. NSG-BLT mice develop a human immune system with a more complete range of human T cells, B cells, dendritic cells (DC), monocytes and macrophages as well as improved engraftment of secondary lymphoid organs. They are capable of mounting adaptive immune responses to infection with Epstein Barr virus (EBV) and human immunodeficiency virus (HIV) [10,11]. A compelling application has been for HIV research: humanized mice support HIV replication after mucosal infection with subsequent CD4^+^ T cell depletion [12,13].

In the current study, we explored the feasibility of using humanized mice for TB research. Toward this end, we investigated whether engrafting human immune cells in NSG-BLT mice elicits 1) cellular responses to Mtb that 2) lead to protection against the pathogen. In an attempt to maximize human immune responses, we engrafted NSG mice expressing HLA-A2.1 molecules (NSG-A2) with HLA-2-positive fetal tissues to enable engrafted HLA-A2-restricted human T cells to recognize murine HLA-A2^+^ phagocytes infected with Mtb. We report here the generation of Mtb-specific human cellular immune responses in NSG-A2-BLT mice, but these responses did not translate into the control of BCG growth or human-like granuloma formation, which might be due to insufficient recruitment of T cells to the lesion and low IFN-γ production.

## Results

### Engrafted human immune cells in NSG-A2-BLT mice mount an acquired immune response to BCG infection

**Figure 1:**
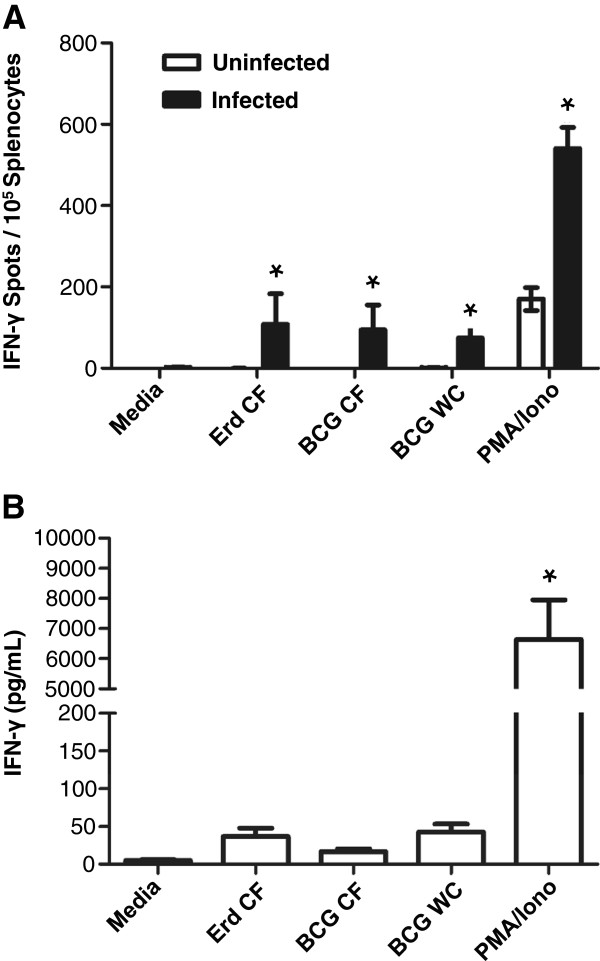
**NSG-A2-BLT mice mount adaptive immune responses to BCG infection.** NSG-A2-BLT mice were infected i.v. with BCG at a dose of 1 × 10^5^ CFU per mouse. One month later mice were sacrificed, and T cell recall response of splenocytes was measured by ELISPOT (n = 6 for infected NSG-A2-BLT mice and n = 2 for uninfected NSG-A2-BLT mice) **(A)** and ELISA (n = 3 for infected NSG-BLT mice) **(B)**. Culture conditions and antigenic stimulation are described in Methods. Abbreviations: CFP, Culture filtrate proteins; WC, Heat-killed whole BCG; PMA, Phorbol 12-myristate 13-acetate; Iono, ionomycin. *p < 0.05.

**Figure 2:**
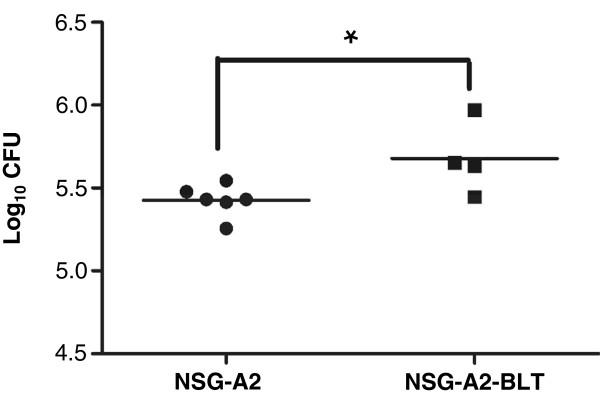
**NSG-A2-BLT mice had a higher BCG load in the lung than A2-NSG mice.** NSG-A2-BLT and A2-NSG mice were infected as indicated in Figure 1, and sacrificed to collect lungs to measure the bacterial load. n = 6 for A2-NSG mice and n = 4 for NSG-A2-BLT mice. *p < 0.05.

To evaluate the utility of NSG-A2-BLT mice as an animal model for TB, we assessed the response of engrafted human immune cells to infection with BCG. NSG-A2 and NSG-A2-BLT mice were infected with BCG intravenously (i.v.) at a dose of 1 × 10^5^ colony-forming units (CFU) and euthanized four weeks post-infection to measure bacterial load in the lung, and human adaptive immune responses. Splenocytes from infected NSG-A2-BLT mice responded to ex vivo restimulation with mycobacterial antigens by producing IFN-γ as measured by ELISPOT, while splenocytes from uninfected NSG-A2-BLT mice did not produce measurable IFN-γ in response to any of these antigens (Figure 1A). Splenocytes from BCG infected mice had a stronger response to activation by PMA/ionomycin than splenocytes from uninfected mice, indicating an expansion of non-antigen-specific Th1 cells from infected NSG-A2-BLT mice. Splenocytes from infected NSG-A2-BLT mice did not produce IFN-γ without antigenic stimulation. Unlike the ELISPOT result, the levels of IFN-γ secreted into media in response to ex vivo antigenic stimulation measured by ELISA were not statistically higher than in the media control (Figure 1B). Splenocytes stimulated with PMA/ionomycin produced large amounts of IFN-γ, indicating the presence of functionally competent T cells capable of secreting IFN-γ. To determine if NSG-A2-BLT mice acquired the capacity to restrict BCG replication, we compared lung bacillary load in NSG-A2-BLT mice and NSG-A2 mice 4 weeks after BCG infection. Rather than demonstrating enhanced control of BCG, the lungs of NSG-A2-BLT mice had higher CFU counts than the non-engrafted NSG-A2 controls (Figure 2). This unexpected result implies that the engrafted human immune cells are unable to kill intracellular bacilli, instead providing an environment more conducive for replication than host murine macrophages.

### BCG-infected NSG-A2-BLT mice develop lesions in the lungs that are not typical granulomas

**Figure 3:**
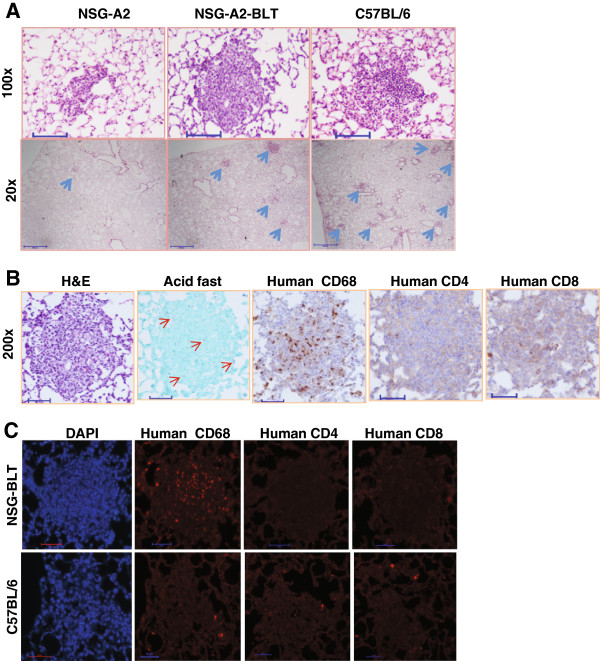
**NSG-A2-BLT mice develop lung lesions in response to BCG infection.** Mice were infected as indicated in Figure 1, and sacrificed for histopathological examination. Lungs were inflated in 10% formalin, and embedded in paraffin for making thin sections. **A**. H&E stains on NSG-A2, NSG-A2-BLT, and C57BL/6 mice (100× and 20×, scale bars = 100 μm and 500 μm, respectively). Blue arrows indicate BCG lesions. **B**. Serial lung sections from NSG-A2-BLT mice infected with BCG were stained for H&E, Acid Fast bacilli, and immunohistochemistry for human CD68, human CD4, and human CD8. Immunostaining was visualized by colorimetric detection. Red arrows indicate BCG bacilli (200×, scale bars = 50 μm). **C**. Serial lung sections from NSG-A2-BLT and C57BL/6 mice infected with BCG were stained with human-specific antibodies against CD4, CD8, and CD68 followed by secondary antibody labeled with Alexa 555, and examined by fluorescence microscopy (200×, scale bar = 50 μm). Images are representative of tissues from four animals.

Human granulomas are not reproduced in murine models of TB [5]. We sought to determine whether engrafted human immune cells develop lesions resembling human granulomas. Lung tissue sections were prepared for H&E, Ziehl-Neelsen (“acid-fast”), and immunostaining for human immune cell markers (Figure 3). H&E staining revealed inflammatory lesions in the lungs of BCG-infected NSG-A2-BLT mice (Figure 3A). No lesions were found in uninfected NSG-A2-BLT mice (data not shown). Non-engrafted NSG-A2 mice infected with BCG had myeloid cell aggregates that were smaller and less frequent than the lesions in NSG-A2-BLT mice. When compared with lesions in immunocompetent C57BL/6 mice infected with BCG in parallel, the lesions of NSG-A2-BLT mice were less frequent but comparable in size, and appeared to be more compact (Figure 3A). Ziehl-Neelsen staining in NSG-A2-BLT mouse lung sections showed that bacilli were scattered throughout the lung, inside and outside areas of inflammation, indicating that the lesions did not prevent spreading of infection. To evaluate human cell recruitment, serial lung sections from NSG-A2-BLT mice were stained with human-specific antibodies against CD4, CD8, and CD68 followed by colorimetric (Figure 3B) and fluorescent detection (Figure 3C). The lesions of NSG-A2-BLT mice contained abundant CD68^+^ human macrophages, but few to no detectable human CD4^+^ and CD8^+^ cells. All three antibodies produced negligible signals in lung sections from BCG-infected C57BL/6 mice, confirming their specificity for human antigens (Figure 3C). No caseous necrosis was observed. Collectively, the lesions induced by BCG in NSG-A2-BLT mice were less well developed than human granulomas.

### Human macrophages promote BCG growth

**Figure 4:**
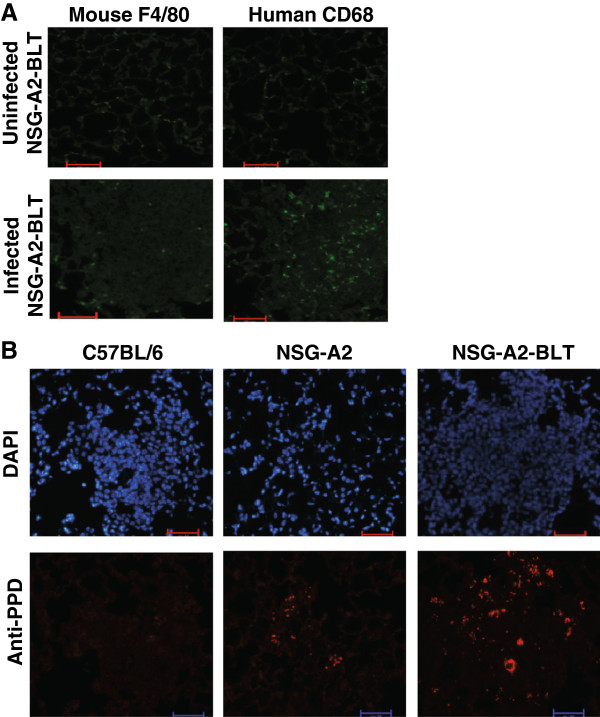
**Human macrophages are the dominant mononuclear cells in the lesions that promote BCG growth. A**. Lung sections prepared from NSG-A2-BLT mice infected with BCG as described in Figure 1 were stained with FITC-labeled anti-human CD68 and FITC-labeled anti-mouse F4/80 (200×, scale bars = 50 μm) **(A)**. Lung sections were stained with anti-PPD antibody followed by secondary antibody labeled with Alexa 555. DAPI was used for counter staining (200×, scale bars = 50 μm) **(B)**. Images are representative of tissues from four animals.

We hypothesized that the lack of protection and higher BCG growth in NSG-A2-BLT mice relative to non-engrafted NSG-A2 mice was due to residual mouse-origin macrophages concealing BCG from human adaptive immune responses despite the expression of human HLA-A2 in both strains. To test that hypothesis, we compared the relative abundance of mouse and human macrophages in the lungs of NSG-A2-BLT mice. Mouse and human macrophages were visualized by staining with anti-mouse F4/80 and anti-human CD68 antibody. Without infection, the frequencies of mouse and human macrophages in the lungs were comparable and low (Figure 4A). On the other hand, human macrophages outnumbered murine macrophages in the lesions of BCG-infected mice (Figure 4A). When lung sections were stained with anti-PPD to visualize BCG bacilli, a large number of BCG bacilli were found in the lung lesions (Figure 4B). This observation suggests that human macrophages recruited to the site of infection support BCG growth better than murine macrophages. In C57BL/6 mice infected with BCG in parallel with NSG-A2-BLT mice, there were much fewer BCG bacilli found in the lung lesions due to efficient adaptive immune responses (Figure 4B). Thus, NSG-A2-BLT mice likely lack efficient T cell effector function at the site of infection.

**Figure 5:**
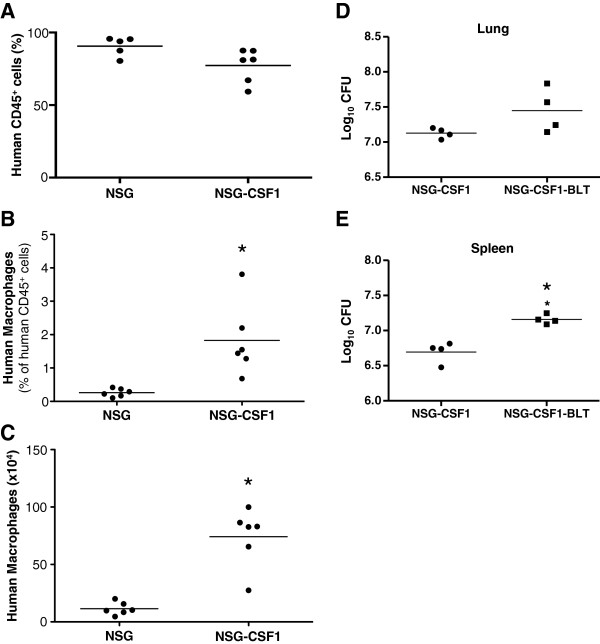
**Transgenic expression of CSF-1 does not improve the protection against BCG in NSG-BLT mice.** NSG-BLT and NSG-CSF1-BLT mice were generated as described in the Methods section. At 16 weeks spleens from NSG-CSF1-BLT mice were recovered and tested for human cell engraftment **(A)** and human macrophage development (**B** and **C**). Human macrophage were defined as CD14^+^/CD33^+^ and the **(B)** percentage and **(C)** number of positive cells are shown. NSG-CSF1 and NSG-CSF1-BLT mice were infected with BCG and bacterial loads in the lung **(D)** and spleen **(E)** were measured by CFU assay **(D)**. N = 4 for NSG-CSF1 and NSG-CSF1-BLT mice. *p < 0.05.

Even though human macrophages were the dominant macrophage population in lesions, murine macrophages were still present (Figure 4A). Murine macrophages could be a major site for BCG replication since they would be less responsive to the effector functions of human T cells. To address that possibility, we used NSG mice transgenically expressing human colony stimulating factor-1 (CSF-1) to generate NSG-CSF1-BLT mice with an expanded human macrophage population. NSG-CSF1-BLT mice had significantly higher levels of human macrophages both in percentages and numbers in the spleen at 16 weeks post-implant (Figure 5A-C). However, BCG load was not reduced in the lungs and spleens of NSG-CSF1-BLT mice, compared to NSG-CSF1 mice, and in fact were higher in the spleen of NSG-CSF1-BLT mice (Figure 5D and E). This suggests that the residual murine macrophages had a minimal impact on the protective immune response mediated by the engrafted human immune cells.

## Discussion

Immunocompetent rodent models fail to accurately reproduce all of the features of human TB, which presents a major obstacle to their use in TB research. Humanized small animal models have shown potential to study functions of human immune cells and tissues in other infectious diseases [14-16]. Thus, we evaluated the utility of NSG-A2-BLT mice for TB research. We show for the first time that a human immune system in mice can mount an antigen-specific T cell response to mycobacterial infection. In addition, human macrophages are recruited to the site of infection forming a macrophage mass, an early phase of granuloma formation. These observations warrant further efforts to refine humanized murine models to study human immunity to TB.

It is encouraging that NSG-A2-BLT mice mounted an antigen-specific T cell response to mycobacterial infection. Previous studies of engrafted human immune cells in mice infected with mycobacteria reported only non-antigen-specific polyclonal T cell activation [17,18]. Priming of antigen-specific T cells requires a multitude of functionalities of both antigen-presenting cells and T cells. DCs are equipped with danger signaling pathways, HLAs and costimulatory molecules, and proinflammatory cytokines that augment antigen presentation [19,20]. It has been shown that NSG-BLT mice mount recall responses to viral infection and immunization with proteins [10,11]. Our report adds one more infection model where NSG-BLT mice can develop a human adaptive immune response to the infectious agent. The elicitation of in vitro recall responses to both secreted Mtb proteins and heat-killed BCG implies that human DCs are capable of processing multiple antigenic forms and presenting Mtb epitopes to naïve human T cells that are capable of priming.

Despite the presence of antigen-specific IFN-γ production by human T cells, NSG-A2-BLT mice were no more capable of restricting BCG replication than non-engrafted NSG-A2 controls. Healthy people and immunocompetent mouse strains are capable of controlling BCG infection, while immunocompromised mice, such as SCID mice, are susceptible to BCG, succumbing to infection around 70 days post-infection [21]. Protective immunity to BCG represents a minimum level of protective immunity against mycobacterial infection, which NSG-A2-BLT mice cannot achieve. Killing of intracellular Mtb requires both innate and adaptive immune components. Human macrophages utilize the vitamin D pathway and autophagy, which are induced after TLR and IFN-γ stimulation [22-24]. While NSG-A2-BLT mice used in this study were fed chow containing vitamin D, the potential for further supplementation to enhance immunity remains to be tested. Regarding adaptive immunity, TB defense depends on the magnitude and quality of the T cell response [25]. Using knockout mice, it has been established that CD4^+^ and CD8^+^ T cells are critical components of anti-mycobacterial immunity with CD4^+^ T cells being more critical than CD8^+^ T cells [26] and mice lacking Th1 cytokines (e.g., IFN-γ, TNF-α) rapidly succumb to Mtb infection [27,28]. These findings are consistent with Mendelian susceptibility to mycobacterial infection in humans [3]. We showed by ELISA that levels of IFN-γ were not significantly induced in response to antigenic stimulation. The observation that PMA/ionomycin stimulated T cells to secrete a large amount of IFN-γ suggests that suboptimal T cell priming and/or expansion of antigen-specific T cells may be responsible for the decreased IFN-γ production capacity of T cells. Since DCs play a critical role in T cell priming [20], more in-depth evaluation of DC functions in NSG-A2-BLT mice may shed light on the lack of protective immunity to infection.

Our data suggest that human macrophages in NSG-A2-BLT mice promoted BCG growth. Heuts et al. [18] similarly reported that lung BCG loads were higher in NSG mice engrafted with human CD34^+^ HSCs than in non-engrafted NSG controls, which they attributed to dysfunctional CD4^+^ T cells. In addition to the potential role of dysfunctional CD4^+^ T cells resulting in an increased bacterial load, we propose that the higher BCG growth in the humanized mice may also lie in the presence of human macrophages. We previously showed that resident alveolar macrophages are the initial target cells for Mtb infection and growth, but are quickly replaced as major Mtb infected cells by macrophages and DCs newly recruited to the lung [29]. In the current study, we show that the lesions were mostly composed of newly recruited human macrophages with few to no mouse macrophages. This homing capability of human macrophages provides niche for BCG growth, as the dominant growth of BCG bacilli took place in the lesions. On the other hand, less developed lung lesions seen in NSG-A2 mice reflect compromised macrophage homing, resulting in limited BCG growth. The lesions of C57BL/6 mice contained few bacteria, indicative of protective acquired murine immune responses. Based on poor T cell recruitment in the lesions and low IFN-γ production, NSG-A2-BLT mice appear to lack optimal functional adaptive immune responses, allowing rapid BCG growth. The higher bacterial load in NSG-A2-BLT mice shown in this study paradoxically demonstrates the functionality of certain aspects of human immune responses including tissue homing and phagocytosis of bacteria. In this regard, NSG-A2-BLT mice may serve as a system to study early events in human Mtb infection prior to the induction of adaptive immunity that is protective. This condition cannot be directly studied in the human host.

Granulomas, a hallmark of immunity to TB, are cellular structures that limit Mtb dissemination and growth [2,30]. In humans, granulomas start as an accumulation of macrophages recruited to the site of infected alveolar macrophages, which are subsequently surrounded by antigen-specific and non-specific T cells [2,30,31]. Analysis of lung tissue sections from NSG-A2-BLT mice revealed that human macrophages were preferentially recruited relative to CD4^+^ and CD8^+^ T cells. The scarcity of human T cells observed at the sites of infection suggests defects in T cell chemotaxis, perhaps due to suboptimal expression of chemokines and/or chemokine receptors. In human granulomas, bacilli are confined in the core of the macrophage mass, and caseous necrosis is often observed. In our study, BCG bacilli were seen in the outer rim of lesions and no caseous necrosis was observed in NSG-A2-BLT mice. In its present iteration, the NSG-A2-BLT model does not recapitulate the granuloma formation that typifies human TB pathology. Recently, two independent studies reported granuloma formation in mice engrafted with human immune cells. Heuts et al. infected NSG mice engrafted with human cord blood as a source of HSC either with 1 × 10^6^ BCG i.v. or with a low dose of Mtb by aerosol. Calderon et al. infected NSG-BLT mice intranasally with 1 × 10^6^ Mtb H37Rv expressing tdTomato [17]. Although the two studies shows certain aspects of human granulomas in the liver, including cellular cuffing, central necrosis, and giant cell formation, neither study provided images of lung lesions that mirror human granulomas. It appears that tissue necrosis is limited to Mtb infection. Like Heuts et al., we did not observe tissue necrosis when BCG was used, whereas Heuts et al. and Calderon et al. observed necrosis with Mtb infection. This is in agreement with our previous report that Mtb more strongly induces macrophage necrosis than BCG [32]. Relatively low frequencies of T cells in our study could be due to the low BCG dose (1 × 10^5^ bacilli) we used. As antigen-specific CD4 T cells are critical to human granuloma formation [33], it would be of value to investigate if enhanced priming of CD4 T cells would enhance T cell migration into inflammatory sites leading to formation of more rigorous, structured lesions.

Humanized mouse models are rapidly evolving but still have significant limitations for TB research [16]. Potential reasons for the failure to express protective immunity include the lack of human hematopoietic growth factors and tissue specific chemokines, and poor lymph node development. A role for relative CSF deficiency in the susceptibility of NSG-A2-BLT mice is intriguing since CSF-1, granulocyte CSF (G-CSF), and granulocyte macrophage CSF (GM-CSF) are all primarily produced by non-hematopoietic cells [34,35]. CSF-1 is the primary regulator of mononuclear phagocyte production and its function has been elucidated with CSF1-deficient *op*/*op* mice and CSF-1 knockout mice [34,36]. The *op*/*op* mice display unstressed osteopetrotic, hematopoietic, tissue macrophages, and abnormal reproductive phenotypes. CSF-1 regulates the morphology and functions of mononuclear phagocytes and is necessary for immunity against intracellular fungal, bacterial, and viral infections in mice reviewed in ([37,38]). Episomal expression of CSF-1 enhances cytotoxicity, superoxide production, phagocytosis, chemotaxis and cytokine production in monocytes and macrophages [39,40]. We show that NSG-CSF1-BLT mice had enhanced relative frequencies of human macrophages. Notwithstanding, NSG-CSF1-BLT mice still could not reduce the bacterial load compared to non-engrafted NSG-CSF1 mice. This excludes the possibility that human CSF-1 deficiency underlies the lack of protective immunity and granuloma formation in infected NSG-A2-BLT mice. Human GM-CSF and G-CSF are deficient in NSG-A2-BLT mice as evidenced by only low levels of circulating human neutrophils [41]. Although the absence of GM-CSF does not affect normal hematopoiesis, GM-CSF^-/-^ mice have numerous defects including a defect in alveolar macrophage maturation, impaired proliferative responses of CD4^+^ T cells to specific antigens after immunization [42,43], and more importantly, increased susceptibility to Mtb infection [44]. Therefore, transgenic expression of human GM-CSF might improve the expression of human immunity to mycobacterial infection in NSG-A2-BLT mice, a hypothesis we are currently testing.

## Conclusions

This study provides supporting data on the potential of humanized mice as an animal model for TB. NSG-A2-BLT mice developed an adaptive immune response to BCG infection. However, this response did not provide protection against infection. Future studies are being directed toward identifying essential immune components in NSG-A2-BLT mice required for more robust human immunity.

## Methods

### Animals and human fetal tissues

Immunodeficient NOD.Cg-*Prkdc*^*scid*^*Il2rg*^*tm1Wjl*^/Sz (NSG) mice and NSG mice transgenically expressing HLA-A2.1 (NSG-A2) or human CSF-1 transgenic (NOD.Cg-*Prkdc*^*scid*^*Il2rg*^*tm1Wjl*^ Tg(CSF1)/Sz , abbreviated as NSG-CSF1, mice were obtained from colonies developed and maintained by Dr. Leonard Shultz at The Jackson Laboratory (Bar Harbor, ME). All animals were housed in a specific pathogen free facility in microisolator cages, and given autoclaved food and maintained on acidified autoclaved water and sulfamethoxazole-trimethoprim medicated water (Goldline Laboratories, Ft. Lauderdale, FL) provided on alternate weeks. All animal use was in accordance with the guidelines of the Animal Care and Use Committee of the University of Massachusetts Medical School and The Jackson Laboratory and conformed to the recommendations in the Guide for the Care and Use of Laboratory Animals (Institute of Laboratory Animal Resources, National Research Council, National Academy of Sciences, 1996). Human fetal thymus and liver tissues were obtained from Advanced Bioscience Resources (Alameda, CA) or StemExpress, Placerville, CA. Protocols involving the use of human tissues were approved by the University of Massachusetts Medical School Institutional Review Board.

### Generation of NSG-BLT mice

NSG-A2 and NSG-CSF-1 transgenic mice at 6–8 weeks of age were irradiated (200 cGy) and surgically implanted under the kidney capsule with 1-mm^3^ fragments of human fetal thymus and liver on the same day as the tissues were received. On the same day as the tissue transplant, CD3-depleted hematopoietic cells derived from autologous fetal liver were injected i.v. into the mice to achieve 1 to 5 × 10^5^ CD34^+^ cells, (we do not positively select) as a source of hematopoietic stem cells (HSC). Human cells were allowed to engraft and to generate an immune system in recipient mice for at least 12 weeks, at which time human hematolymphoid engraftment was validated by flow cytometry on peripheral blood as described previously [45]. The thymic implants in the NSG-BLT mice grew substantially from the initial size of 1 mm^3^ to over 5 mm^3^, and histological analysis revealed that the human thymic tissue was heavily populated with human CD45^+^ lymphocytes. Spleens of NSG-BLT mice contained human B cells and T cells (both CD4^+^ and CD8^+^ single-positive), macrophages (CD14^+^/CD33^+^) and both conventional (CD11c^+^) as well as plasmacytoid (CD123^+^ ) DC. Successfully engrafted mice based on a minimum of 20% human CD45^+^ cells in peripheral blood were then distributed into various groups based on engraftment levels.

### BCG infection

*M. bovis* BCG Pasteur was grown in Middlebrook 7H9 basal medium (DIFCO; Beckton Dickinson, Sparks, MD) supplemented with OADC (oleic acid, albumin, dextrose, and catalase). BCG bacilli were washed with PBS containing 0.05%. Tween 80 (PBS-T80), aliquoted in freezing vials and stored at -70°C until use. For determination of bacterial concentration in the stock, one vial was thawed after 24 hours and the concentration of bacilli was determined by serial dilution followed by plating on 7H11 agar supplemented with OADC. For infection, aliquots of frozen BCG stock in PBS-T80 were thawed and sonicated for 1 min in a cup-horn sonifier (Branson Ultrasonics Corporation, Danbury, CT) to remove clumps. Mice were infected i.v. with BCG at 1 × 10^5^ CFU per mouse.

### ELISPOT/ELISA assay

IFN-γ-secreting cells were quantified using an IFN-γ Ab ELISPOT pair (Mabtech, Nacka Strand, Sweden), MultiScreen-IP ELISPOT plates (Millipore, Billerica, MA), streptavidin-alkaline phosphatase (ALP; Mabtech), and 5-bromo-4-chloro-3-indolyl phosphate/nitro blue tetrazolium, BCIP/NBT (Sigma-Aldrich). The membranes of ELISPOT plates were treated with 15 μl 70% ethanol followed by 8 washes with water and then coated with detection Ab diluted 1:250 in PBS overnight at 4°C. The plates were washed with PBS and the membrane blocked with complete cell culture medium (RPMI1640 with 10% heat inactivated FCS, 2 mM L-glutamine, 50 μM 2-mercaptoethanol, 100 U/ml penicillin, 100 μg/ml streptomycin sulfate) for a minimum of 2 h at room temperature. Splenocytes, plated at 1 × 10^5^ per well in 200 μl, were stimulated with Mtb antigens including Mtb Erdman culture filtrate protein (CFP), BCG CFP, and BCG heat-killed whole cell (WC). All Mtb antigens were used at 2 μg/ml. A combination of Phorbol 12-myristate 13-acetate (PMA**,** 20 ng/ml) and ionomycin (1 μM), both purchased from Sigma, was used for non-specific T cell stimulation. After 44 h incubation (37°C, 5% CO_2_) cells were washed off the membrane with PBS and biotinylated detection Ab (1:200 in PBS-T20) was added and incubated for 2 h at room temperature. The wells were washed with PBS-T80 and then PBS before incubation with streptavidin-ALP for 1 h at room temperature followed by PBS wash and addition of BCIP/NBT substrate. IFN-γ spots were developed for 7 to 12 min before a final wash with water. The membrane was dried overnight at room temperature. Analysis was performed with an automated ELISPOT reader (ImmunoSpot, Cellular Technology Limited, Shaker Heights, OH), using CTL ImmunoSpot Academic Software version 4.0. Results were calculated as spots per 10^5^ splenocytes, and mean numbers of spots from duplicate wells of each stimulation are reported.

### ELISA

Splenocytes were plated in 48-well plates at 2 × 10^6^ cells in 1 ml of complete media. Cells were stimulated with Mtb antigens and PMA/ionomycin as described above for 4 days and the supernatants were collected and stored at -70°C until use. Secreted IFN-γ levels were assayed by ELISA according to the manufacturer’s protocol (R&D Systems, Minneapolis, MN).

### CFU assay

Lungs were harvested and then homogenized in PBS-T80 using a Bullet Blender homogenizer (Next Advance, Inc., Averill Park, NY). The homogenates were serially diluted, plated in duplicate on Middlebrook 7H11 agar (DIFCO), and cultured at 37°C for 3 weeks. Colonies were counted using a dissecting microscope at day 13 and 21 after plating.

### Immunohistochemistry

Lungs were inflated and fixed with 10% buffered formalin for 24 h and then processed for staining. Paraffin embedded tissues were sectioned and mounted on slides. Mounted slides were stained with hematoxylin & eosin (H&E) for histopathology, and Ziehl*–*Neelsen staining for Mtb detection*.* Immunostaining of slides was performed with anti-human CD4 (Vector Laboratories, Burlingame, CA, 1:100), anti-human CD8 (Dako, Carpinteria, CA, 1:80), anti-human CD68 (Dako, 1:500), anti-mouse F4/80 (AbD Serotec, Raleigh, NC, 1:50) and anti-PPD (Abcam, Cambridge, MA, 1:100). Enzymatic antigen retrieval was performed prior to immunostaining and Ab was detected with Dako Envision + Dual link System-HRP (Dako). Fluorescent detection of primary Abs was performed using Alexa Fluor 555 goat anti rabbit IgG (Life technologies, Grand Island, NY) at a 1:500 dilution for anti-PPD Ab, and Alexa flour 488 or 555 Donkey anti-mouse IgG (Life technologies) at 1:500 for anti-human CD4, CD8, and CD68, and anti-mouse F4/80 Abs. Lung sections were stained with ProLong Gold antifade reagent with DAPI (4′,6-Diamidino-2-Phenylindole, dihydrochloride) (Life technologies) as a counter stain. All staining was performed by the Diabetes and Endocrinology Research Center histopathology core facility at University of Massachusetts Medical School. Stained lung sections were analyzed with a Nikon Eclipse E400 microscope (Nikon Instruments, Melville, NY) using Spot Advanced v.4.6 software (Diagnostic Instruments Inc., Sterling Heights, MN).

### Statistical analysis

Statistical analysis was performed using Graph Pad Prism v.5.02 (Graphpad Software Inc., La Jolla, CA) software. Unless otherwise stated, data from independent experiments are shown as mean ± SEM. Comparisons between groups were evaluated with Student t-test or One-way ANOVA test followed by Dunnett's Multiple Comparison Test. Bacterial load data were transformed using natural logarithms to better approximate normally distributed errors. A p value of 0.05 or lower was regarded as statistically significant.

## Abbreviations

BCG: Bacillus Calmette-Guerin; CSF: Colony stimulating factor; DC: Dendritic cells; EBV: Epstein Barr virus; G-CSF: Granulocyte CSF; GM-CSF: Granulocyte macrophage CSF; HIV: Human immunodeficiency virus; HSC: Hematopoietic stem cells; IFN: Interferon; IL: Interleukin; Mtb: *Mycobacterium tuberculosis*; NSG mice: NOD/SCID/IL-2Rγ^-/-^ (NSG) mice; BLT: Bone marrow-liver-thymus; TB: Tuberculosis; TNF: Tumor necrosis factor; SCID: Severe combined immune deficiency.

## Competing interests

The authors declare that they have no competing interests.

## Authors’ contributions

JL led study design, carried out BCG infection, immunoassays, CFU assay and manuscript preparation. MB generated NSG-BLT mice, participated in study design, characterized NSG-BLT-CSF-1 mice, and manuscript preparation. DG participated in study design/data analysis/interpretation, and provided expertise in humanized mice. LS generated NSG-CSF-1 mice and manuscript revision. HK conceived of the study, participated in study design, and manuscript preparation. All authors read and approved the final manuscript.
